# Blood glucose monitoring devices for type 1 diabetes: a journey from the food and drug administration approval to market availability

**DOI:** 10.3389/fendo.2024.1352302

**Published:** 2024-03-15

**Authors:** Rahul Mittal, Nicole Koutras, Jonathan Maya, Joana R. N. Lemos, Khemraj Hirani

**Affiliations:** ^1^ Diabetes Research Institute, University of Miami Miller School of Medicine, Miami, FL, United States; ^2^ Herbert Wertheim College of Medicine, Florida International University, Miami, FL, United States

**Keywords:** type 1 diabetes, glycemic control, food and drug administration, electrochemical sensors, continuous glucose monitoring devices, regulatory approval

## Abstract

Blood glucose monitoring constitutes a pivotal element in the clinical management of Type 1 diabetes (T1D), a globally escalating metabolic disorder. Continuous glucose monitoring (CGM) devices have demonstrated efficacy in optimizing glycemic control, mitigating adverse health outcomes, and augmenting the overall quality of life for individuals afflicted with T1D. Recent progress in the field encompasses the refinement of electrochemical sensors, which enhances the effectiveness of blood glucose monitoring. This progress empowers patients to assume greater control over their health, alleviating the burdens associated with their condition, and contributing to the overall alleviation of the healthcare system. The introduction of novel medical devices, whether derived from existing prototypes or originating as innovative creations, necessitates adherence to a rigorous approval process regulated by the Food and Drug Administration (FDA). Diverse device classifications, stratified by their associated risks, dictate distinct approval pathways, each characterized by varying timelines. This review underscores recent advancements in blood glucose monitoring devices primarily based on electrochemical sensors and elucidates their regulatory journey towards FDA approval. The advent of innovative, non-invasive blood glucose monitoring devices holds promise for maintaining stringent glycemic control, thereby preventing T1D-associated comorbidities, and extending the life expectancy of affected individuals.

## Introduction

Type 1 diabetes mellitus (T1D) impacts 9.5% of people globally and has an increasing incidence worldwide (1–3). T1D is associated with extensive complications, which fall into three main categories: macrovascular, microvascular, or metabolic (4). In children with T1D, the most common cause of death is related to metabolic issues in children with T1D, including diabetic ketoacidosis (DKA) and hypoglycemia (5, 6). In adults, however, death is more commonly due to macrovascular and microvascular problems (5). Microvascular complications include diabetic neuropathy, nephropathy, and retinopathy whereas macrovascular complications include peripheral vascular disease, stroke, and cardiovascular disease (1, 7).

Due to these complications, patients with T1D have shorter life expectancies than those without T1D. The standardized mortality rate (SMR) for all-cause mortality is 4.5 in individuals with T1D when compared with those who do not have T1D (8). Cardiovascular disease is the largest contributor to the increased mortality in T1D patients and accounts for 37.5% of all deaths due to T1D and almost 50% of the years of life lost (9). T1D patients have an SMR of 6.6 due to cardiovascular disease alone when compared to the general population (8). Endocrine and metabolic diseases are the second largest contributor to T1D mortality and comprise 20.7% of all deaths due to T1D and almost 30% of all years of life lost (9).

Along with increased mortality, T1D has been associated with co-morbidities. Diabetic retinopathy is one of the most common complications of T1D, with a prevalence rate of 20-25% and is the leading cause of acquired blindness (10–12). After 15 to 20 years of living with T1D most adults will have some form of diabetic retinopathy. Approximately 20% to 30% of those cases will lead to blindness (13). Diabetic neuropathy is another common complication of T1D with conditions including gastroparesis, carpal tunnel syndrome, and nerve palsies (14). When diabetic neuropathy is in conjunction with peripheral vascular disease it can cause diabetic foot ulcers, which may require amputation (15, 16). Increased attention to glycemic control is necessary for individuals diagnosed with diabetic retinopathy or diabetic neuropathy to better control their symptoms and prevent further complications.

Unfortunately, it has been shown that 80% of adults with T1D have suboptimal glycemic control, with a mean HbA1c of 8.8% while the American Diabetes Association (ADA) recommends an HbA1c of <7.0% in nonpregnant adults (17). Even the less stringent HbA1c goals recommended by the ADA for those with limited life expectancy or those for whom the benefit of glycemic control does not outweigh the harms is <8.0% (18, 19). Children have also been shown to have suboptimal glycemic control demonstrating HbA1c measurements of 7.63% while the ADA and the International Society for Pediatric and Adolescent Diabetes (ISPAD) recommend an HbA1c goal of <7.0% for children (20, 21). Less stringent goals for children have been set at <7.5% and <8.0% for certain populations (20, 22).

Along with having a large disease burden for the patient, T1D poses a substantial economic constraint to the healthcare system in the United States (23). According to recent data from the American Diabetes Association (ADA), the economic burden of diabetes in the U.S. was estimated at $327 billion in 2017, with approximately $15 billion allocated specifically to T1D-related expenses (24). Pharmacy costs make up over half of the monthly diabetes-related cost and are approximately $440 per person per month (PPPM) (25). Although hospitalizations are relatively rare, they have a large financial burden and comprise 11.5% to 13.9% of the total monthly cost of T1D.

The use of continuous glucose monitoring (CGM) devices among people with T1D is on the rise. These devices are associated with lower levels of HbA1c in this population, indicating better glycemic control (26). CGM device measurements of the amount of time spent within the target blood glucose range correlate negatively with HbA1c (27). CGM measurements of time above range (TAR) > 180mg/dL have been shown to correlate positively with a high blood glucose index whereas time in range (TIR) has a negative correlation with high blood glucose index (22). For every 10% change of TIR there was shown to be a 0.7% change in HbA1c.It has been shown that participants with HbA1c ≤7.0% had a median TIR of 72.1% while those with an HbA1c ≥8.5% had a median TIR of 35.5% (27).

The growing adoption of CGM devices has spurred significant progress in the technology of blood glucose monitoring devices, with a specific emphasis on the development of non-invasive and minimally-invasive methods (28–32). These approaches offer several advantages over more traditional and invasive procedures, such as finger sticks. They provide patients with reduced pain and discomfort, along with lowering the risk of infection and tissue damage (33). Non-invasive devices predominantly utilize sensors placed on the skin’s surface to measure blood glucose concentrations, obviating the necessity for needle penetration into the body (34). Minimally-invasive devices either sample interstitial fluid using a less invasive needle or explore alternative bodily fluids such as tears for blood glucose measurement, presenting a less intrusive option compared to traditional needlestick methods (35).

**Figure 1:**
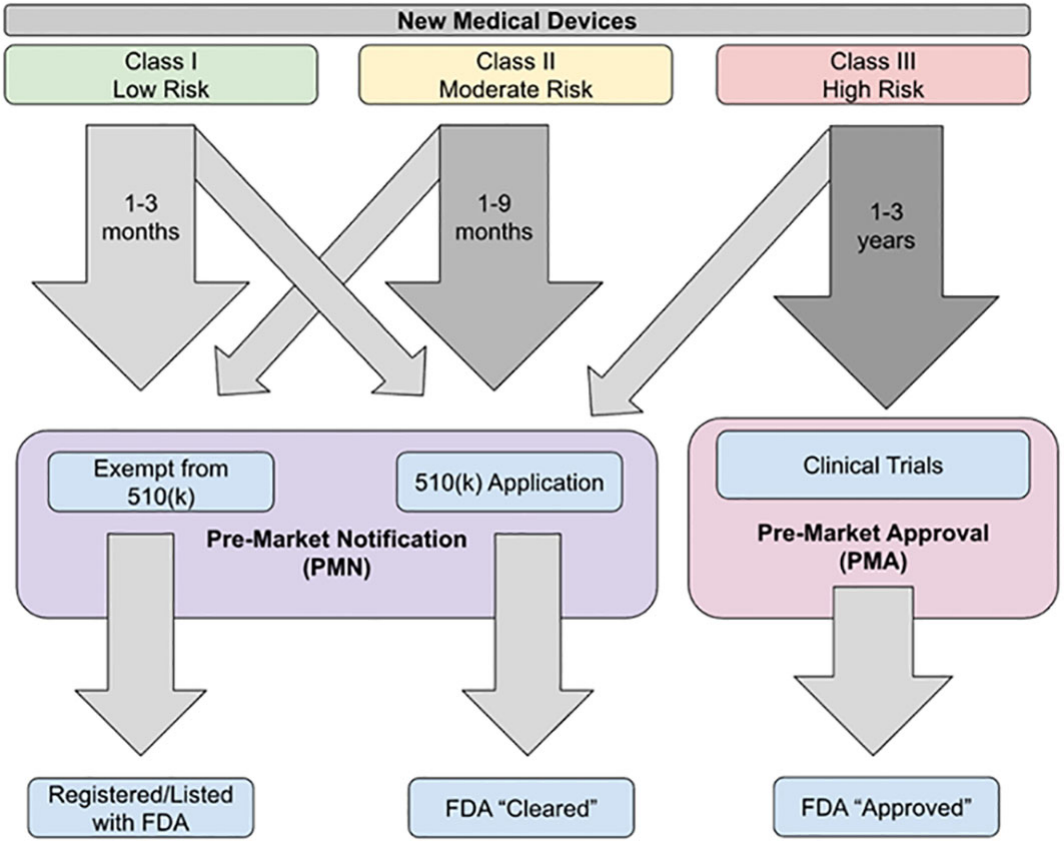
A schematic representation of FDA approval procedure.

The objective of this narrative review article is to summarize the recent advancements in blood glucose monitoring devices primarily based on electrochemical sensors (**Table 1**). We provide an overview regarding how these blood glucose monitoring devices are approved and regulated by the U.S. Food and Drug Administration (FDA) before they are available in the market (**Figure 1**). Ensuring rigorous glycemic control through the use of these blood glucose monitoring devices is essential for the effective management of T1D and the prevention of potentially life-threatening co-morbidities.

**Table 1:** Overview of blood glucose monitoring techniques based on electrochemical sensors.

Device Name	Year of FDA Approval	Key Features	PMN/PMA Number
Modified Clark Enzyme Electrode	N/A	• Provides a larger surface area for the working electrode	N/A
Senseonics Eversense	2018	• Convenience for users via mobile app • Overcomes miniaturization challenges	P160048
MiniMed 780G™ and Guardian™ 4 Sensor	2018	• Minimally invasive • Demonstrated safety • Improvements in user’s glycemic control • Reduction in T1D burden • FDA approved	P160007
Dexcom G6	2018	• Minimally invasive • Improvements in user’s glycemic control • Increased capturing of hypoglycemic events • FDA cleared	K182041
Dexcom G7	2022	• Reduced warm-up time • Enhanced accuracy • Smaller and more discreet design • Integrated smartphone app connectivity • Extended wear duration • Advanced alert system • Share feature for remote monitoring • Calibration-free operation	K213919
*FreeStyle Libre 3*	2022	• Higher accuracy • Real-time glucose monitoring • Minute-by-minute updates • Smaller and more discreet • Enhanced connectivity • Longer sensor wear time • Alarm functionality • No fingerstick calibration • Water-resistant • Improved adhesive	K212132
Raman Spectroscopy	N/A	• Non-invasive • Demonstrated safety • Calibration stability for 15 days • Accurate for a variety of skin tones	N/A
Zinc Oxide Micropipette Tip	N/A	• Uses an affordable plastic to reduce cost • Faster electron transfer	N/A

## The United States food and drug administration regulatory process

Since 1976, the FDA has been responsible for ensuring the safety of medical devices sold to consumers. This responsibility was established when the Federal Food, Drug, and Cosmetic Act was amended to include medical devices (36). According to this act, a device is any “instrument, apparatus, implement, machine, contrivance, implant, *in vitro* reagent, or other similar or related article, including any component, part, or accessory” which meets the conditions of being: 1) recognized in the official National Formulary, the United States Pharmacopeia, or any supplement to them; 2) intended for use in the diagnosis of disease or other conditions, or in the cure, mitigation, treatment, or prevention of disease in humans or other animals; or 3) intended to affect the structure or any function of the body of humans or other animals, and does not achieve its primary intended purposes through chemical action within or on the body of humans or other animals nor is dependent on being metabolized to achieve its primary intended purposes (37). Medical devices are regulated by the Center for Devices and Radiological Health (38).

Most medical devices on the market are consecutive iterations of previous devices that have already been approved. However, if a device is completely new, it usually goes through the process of being built as a prototype and patented followed by evaluation on preclinical animal models (36). This process is cyclical with many different changes being required as the testing procedure continues and can take 2-3 years as well as $10-20 million in cost. This process is only the preclinical stage for a completely novel device and is required before it can be used in clinical trials (36).

The FDA classifies medical devices into one of three categories (**Table 2**). Class I devices are associated with low risk of injury or illness (such as toothbrushes), Class II have a moderate risk of injury or illness (such as sutures), and Class III have a high risk of injury or illness (such as pacemakers) (36, 39). Class III devices have the strictest requirements, whereas Class I and II devices do not require extensive preclinical or clinical trial data. All new devices which do not have a predecessor that has been FDA approved are classified as a Class III device unless the company applies for an exemption due to the device being low risk; if granted then the device is classified as a “*de novo*” device (36).

**Table 2:** Summary of medical device classification.

Classification	Risk	Pathway	Examples
Class I	Minimal	No FDA approval needed Device registered with FDA website 30-90 days	Toothbrushes
			Adhesive Bandages
			Sanitary Pads
			Tongue Depressors
Class II	Moderate	FDA clearance required “Pre-Market Notification” (PMN) 510(k) Application 1-9 months	Continuous Blood Glucose Monitors
			Ultrasound
			Sutures
			Blood Pressure Cuffs
Class III	High	FDA approval required Requires clinical trials “Pre-Market Approval” (PMA) 1-3 years	Pacemakers
			Defibrillators
			Implanted prosthetics
			Cochlear implants

The FDA has three main pathways for approval of medical devices: pre-market approval (PMA), pre-market notification (PMN), and humanitarian device exemption (HDE) (**Figure 1**). Blood glucose monitoring (BGM) devices are primarily approved through either the PMA or PMN pathways (36).

The PMA pathway is used when there is not an FDA-approved pre-existing device that is equivalent to the new device. This is the pathway that must be used for approval of Class III devices unless they have been reclassified as *de novo* devices. There must be sufficient evidence to show that the device is safe for use and effective. In order to conduct this research, investigators need to obtain an investigational device exemption and institutional review board (IRB) clearance, which can lead to the approval process for research taking upwards of a year. This length of time has led to much of the testing being conducted outside of the United States (36). The level of evidence required is usually Level I or Level II evidence. Once the application has been submitted and approved, the device is considered to be “FDA approved” (39).

The PMN pathway, also known as the 510(K) application, is used when there is already an existing device on the market that is similar to the new device. This is a fast-tracked process that requires demonstration that the new device is substantially equivalent to the device that is currently approved and is available in the market (36). This is the primary approval pathway for Class I, Class II, and *de novo* devices. For this pathway, preclinical data is usually sufficient and clinical trial data is not generally required (39). There are some critiques of this pathway, including concerns over “serial predicates’’ in which a device is approved using an existing device as its predicate, even though that existing device was approved using another device as its predicate; such serial predicates may be traced back through several generations of devices. This can leave a substantial gap between the newest device approved and the last device to go through rigorous testing, with many predicate devices in between (36). Once a device is approved through the PMN pathway, it is considered to be “FDA cleared” (39).

Prior to 2018, the FDA required CGM devices to be classified as Class III devices, meaning that they were required to go through the more stringent PMA pathway. In 2018, however, the Dexcom G6 was classified as a Class II device and has criteria known as special controls. This has allowed subsequent CGM devices to go through the less strict 510(K) pathway (40–42).

The FDA also has requirements for self-monitoring over-the-counter blood glucose devices that are intended to protect the lay person using these devices (42, 43). The lay person must be able to prick their own finger and perform the blood glucose measurement using only the directions on the packaging of the device. As well as being accessible, the device must also demonstrate accuracy when it comes to these measurements. The FDA requires 95% of the readings to be within ± 15% of the comparator results, and 99% of the measurements to be within ± 20% of the comparator results. These requirements differ from the requirements set forth by the International Standards Organization document ISO15197, which is used in most countries in the European Union and Canada as the standard for blood glucose monitoring devices (42).

## Comparison of regulatory processes between the U.S. FDA and European Union

In comparison to the FDA in the United States, the European Union has several key differences in the regulatory and approval process for medical devices and pharmaceuticals. A key difference between the EU and the U.S. in terms of medical device regulation is the absence of a centralized competent authority in the EU (44). This contrasts with the role of the U.S. FDA, which acts as a centralized body overseeing market approvals. In the U.S., once a device receives FDA approval, there is no specific time limit on how long it can remain in the market, provided it is not subject to a recall due to safety concerns or other issues. In contrast, the EU has a different approach. Medical devices in the EU are subject to a limited validity period, typically around five years. After this period, these devices must undergo a reassessment procedure to renew their market approval (44). A comparison between the regulatory process of U.S. FDA and EMA is summarized in **Supplementary Table 1.**

Approximately a decade ago, the perception prevailed that European regulatory bodies were more expedient in approving medical devices, particularly in the realm of CGM devices integrated with insulin pumps, compared to the U.S. FDA. This perceived swiftness in Europe could be attributed to a variety of factors, including differing regulatory frameworks and approaches to medical device approval (44). The European system, governed by the Conformité Européenne (CE) mark, often allowed for a quicker path to the market for medical devices. This process was seen as less cumbersome compared to the U.S. FDA’s stringent Premarket Approval (PMA) or 510(k) clearance procedures, especially for novel medical technologies. The European Medicines Agency (EMA) and various national regulatory agencies in Europe had an approach that many believed to be more facilitative for rapid introduction of new medical devices (44).

However, in recent years, there appears to be a shift in this dynamic. More often, medical devices, including CGM systems and insulin pumps, are receiving approval in the U.S. before being approved in Europe. Several factors might contribute to this change. A significant number of medical device manufacturers are based in the U.S. These companies may prioritize the U.S. FDA approval to first enter their domestic market, which is one of the largest for medical devices globally (44). In addition, the U.S. FDA has made efforts to streamline its approval process, especially for successor devices or those that represent incremental innovations over existing technologies. This change is partly in response to criticisms of the U.S. FDA’s previously lengthy and complex approval processes and is intended to foster innovation while maintaining stringent safety standards. Furthermore, the introduction of the Medical Device Regulation (MDR) in the EU, fully applied from 2021, has brought more stringent requirements for medical devices, including more rigorous clinical evidence and post-market surveillance (44). This shift could potentially slow down the approval process in Europe compared to the past. It is also possible that manufacturers may also be adapting their global strategy, considering various factors such as market size, healthcare reimbursement policies, and the competitive landscape, which could influence where and how they seek regulatory approvals. The shift in the process of regulatory approval reflects the ongoing efforts of global healthcare industry to balance innovation with patient safety and device effectiveness.

## Biosensor devices

In the past few years, glucometers, the standard tool for determining blood glucose level, have become less successful and more costly. This has led to the increased popularity of biosensors, analytical instruments with a biological sensing aspect to them, that continuously monitor blood glucose rather than only at a single point in time like a glucometer (45). Biosensors come in various forms, each of which have been optimized for continuous glucose monitoring: electrochemical, optical, enzymatic, non-enzymatic, noninvasive, and real-time biosensors (46, 47) (**Table 1**).

Minimally invasive and non-invasive blood glucose monitoring devices have been the focus of research in recent years (33, 48–50). These devices are able to monitor blood glucose levels with minimal to no pain or discomfort, or the invasiveness that is associated with traditional methods of measuring blood glucose (33). Minimally-invasive devices, such as CGMs, sample the interstitial fluid to determine the blood glucose concentration (51, 52), while non-invasive devices use technology such as spectroscopy to measure blood glucose from the surface of the body without the need for a needlestick (35). Traditional methods of blood glucose monitoring, by comparison, are more invasive and require whole blood, plasma, or serum for the measurement (33).

### Novel modification to the Clark enzyme electrode

An implantable enzyme-electrode sensor remains the most popular interstitial fluid analysis technique for CGM and was one of the first developed for consumers (53). This electrochemical biosensor is widely used but has limited sensitivity, due to a narrow working electrode (WE) area, thus dampening the accurate detection of hypoglycemia. To address this limitation, a new cylindrical, flexible enzyme-electrode with a larger WE surface area has been proposed (47). By utilizing a cylindrical substrate, the sensor overcomes the diameter constraints imposed by conventional pin-like sensors and allows for formation of a WE over not only the radius, but also along the axis of the cylindrical substrate, thus bypassing the diameter restriction. Glucose microsensors were developed by attaching an oxidase enzyme to the tip of this Clark-type oxygen microelectrode, which ranges in size from 15-40 micrometers. These sensors have proven to be rapid and highly sensitive in detecting analyte concentrations, including glucose. However, further research and development is warranted to implement the Clark enzyme electrode in a device.

One main limitation of the modified Clark enzyme electrode is its dependence on oxygen. The blood plasma concentration of dissolved metabolites, for example glucose, is measured by the oxygen electrode with a platinum cathode covered by an oxygen-permeable membrane. To measure glucose specifically, glucose oxidase is immobilized in a gel layer, allowing for the catalysis of glucose, oxygen, and water to yield gluconic acid and hydrogen peroxide. The resulting electrical current is proportional to the glucose concentration.

### Senseonics eversense

Various other techniques are being investigated for glucose sensor development, such as infrared spectroscopy, which faces many limitations and challenges when it comes to frequent calibrations, poor selectivity, limited sensitivity, and miniaturization difficulties (54, 55). One proposed approach, the fluorescence-based device Eversence, designed by Senseonics was developed and FDA approved on June 21, 2018 through the PMA pathway (56). Fluorescence glucose testing assesses signal intensity and duration of decay, and the lifetime of fluorescence differs for each analyte evaluated, thus distinguishing substances (57). This 90-day implanted sensor, after measuring glucose levels, sends information to a mobile app to alert users when there are dangerous fluctuations in blood glucose.

The FDA approved the Eversence device after a clinical study of 71 individuals aged 18 and over with T1D and T2D that reviewed the device’s effectiveness by comparing readings obtained by the device to a laboratory glucose analyzer (58). The mean absolute relative difference (MARD) was found to be 11.1% and 81% of hypoglycemia events were detected within 30 minutes. No serious adverse events were reported during the study (58). While adverse effects related to inserting and wearing the device were observed, such as allergic reactions, bleeding, and bruising, the FDA ultimately granted approval of the device due to the benefits of detecting aberrant blood glucose levels outweighing the risks of not doing so (59, 60).

However, several limitations have been identified with the Senseonics Eversense device. A primary constraint is associated with the device removal process, which necessitates a skin incision for dissection to access the sensor beneath the tissue and fibrous capsule. This procedure can pose significant challenges for certain patients, potentially requiring a minor surgical intervention to facilitate the incision and sensor replacement.

Additionally, the Eversense CGM system does not display glucose readings for up to 24 hours after the device is implanted, due to damage to the surrounding tissue from the sensor. This subsequently causes less sensitivity and accuracy for several days. Errors in calibration also affect the sensor’s accuracy, and there is likely to be a period of time where the device does not sense glucose at its full capacity, until its next recalibration (61).

The company that designed the Eversence device, Glysens, has been developing a new long-term CGM device called the “Eclipse” with multiple electrochemical glucose and oxygen electrodes that measure glucose at five-minute intervals, providing a more accurate reading and remaining significantly more stable between recalibration periods. This device has been functioning well, maintaining accuracy for more than one year during both animal and human clinical trials. Moreover, when a new sensor is implanted in the same site as a fibrous capsule from the previous sensor, it has no effect on the functioning of this device (61).

### MiniMed™ 780G and guardian 4™ sensor

CGM devices are often used in conjunction with automated insulin delivery (AID) devices that use the CGM data to maximize percentage TIR and reduce the amount of time patients spend in a hypoglycemic or hyperglycemic state (62). AID systems using CGM technology have previously demonstrated improvements in HbA1c and percentage TIR in randomized control trials when compared with fingerstick blood glucose monitoring (63–65). Improvements in percentage TIR have been shown to increase TIR by 3.6 hours per day when using AID and CGM technology compared to traditional fingerstick measurements (63). Participants using AID and CGM devices were also shown to have a reduction in HbA1c of 1.42% compared to traditional methods (65).

The MiniMed™ 670G with the Guardian™ sensor 3 was shown to be safe and effective in a 90-day multicenter single-arm study composed of adolescents and adults (66). Safety was demonstrated by having zero adverse or unexpected device effects and zero episodes of DKA during the study period. The study found statistically significant reductions in HbA1c in the adolescent group, the adult group, and overall; the overall reduction in HbA1c was 0.5% during the 90 days (66). There were also statistically significant improvements in %TIR for adolescents, adults, and overall, with the overall %TIR rising from 68% to 72.1% over the study period. The overall MARD was 10.6% (62). The MiniMed™ 670G with the Guardian™ Sensor 3 is the predecessor to the newer MiniMed™ 780G advanced hybrid closed-loop system with the Guardian™ 4 Sensor (66).

In a 3-month multi-center, single-arm, non-randomized study, the MiniMed™ 780G advanced hybrid closed-loop system with the Guardian™ 4 sensor was shown to be safe and reduced the management burden of T1D in both adults and children (62). Safety was shown by having zero serious adverse effects including diabetic ketoacidosis and severe hypoglycemia. Reduction in T1D management burden was shown by having minimal advanced hybrid closed loop system exits, with an average of 0.1 exit per day in both the pediatric and adult groups (62). Percent time below range (%TBR) <54 mg/dL (level 2 hypoglycemia) was 7.8 minutes per day for participants ≤15 years old and 4.8 minutes per day for participants >15 years old. This demonstrates a very low amount of time spent in level 2 hypoglycemia and is a 0.4% reduction (6 minutes/day) compared to using the Guardian™ sensor 3. There have been further improvements from the Guardian™ Sensor 3 with regard to the number of daily blood glucose measurements (BGMs). The number of BGMs in adults decreased from 4.0 ± 1.0 per day to 0.8 ± 0.9 per day when going from the Guardian™ sensor 3 to the Guardian™ 4 sensor. In children this number went from 4.2 ± 1.2 per day to 0.8 ± 0.9 per day (62). The MiniMed 780G and Guardian™ sensor 3 was FDA approved through the PMA pathway on March 8, 2018 (67).

This device has many advantages as demonstrated by its safety, improvements in user’s glycemic control, reduction in T1D burden, and FDA approval (63, 67). Disadvantages include the invasive nature of this device and lag time. Although it is minimally invasive, it still requires a needle to sample the interstitial fluid, which can be uncomfortable for users. There is also a lag in time between changes in blood glucose and the device’s recognition of this change. This is due to the fact that the device is minimally invasive and therefore samples the interstitial fluid instead of the blood (33).

### Dexcom G6

Dexcom G6 is a minimally-invasive CGM that has previously been shown to be efficacious in individuals with T1D by increasing TIR by 3.5 hours per day, with the greatest improvements in %TIR in those who use more of the device’s additional features (68, 69). Additional features include more specific blood glucose alarms, Dexcom CLARITY™ software for analysis, remote monitoring, and a notification system that announces the user’s blood glucose and trends. Dexcom G6 always notifies users when blood glucose levels are low, however it also has a smartphone app which allows users to receive more specific notifications about their blood glucose. These additional alarms include warnings about high blood glucose and soon to be low blood glucose (blood glucose ≤55 mg/dL predicted in the next 20 minutes). Users can adjust the thresholds for these alarms with high blood glucose ranging from 120-400 mg/dL and low blood glucose ranging from 60-100 mg/dL (69). Dexcom G6 was FDA cleared through the PMN (510K) pathway on October 26, 2018 (70).

In recent years CGM devices have gained popularity in clinical trials evaluating the efficacy of diabetes medications. Out of all diabetes medication clinical trials from 2013-2019, 9% used CGM devices compared to 2.7% from 2000-2006 and 5.6% from 2007-2012 (71). This is, in part, due to the significant increase in hypoglycemic events that the devices are able to capture compared to using finger stick measurements, especially at night (72).

In a 12-week, phase 4 multicenter, randomized, active-controlled, parallel group, open-label study, Dexcom G6 was used to measure TIR when comparing two basal insulin (BI) analogues, insulin glargine 300 U/ml (Gla-300) and insulin degludec 100 U/ml (IDeg-100) in adults with T1D (73). The CGM data was used to measure hypoglycemic events, %TIR, %TAR, and %TBR in this study to compare the two BIs. In addition, self-measured plasma glucose (SMPG) was also measured and compared against the CGM data to compare rates of hypoglycemic events (<70 mg/dL). Dexcom G6 was shown to capture 2-6 times more hypoglycemic events in patients with T1D compared to SMPG during the same time period. The most prominent difference was with nocturnal hypoglycemic events (73). This is the first large RCT to use CGM data to assess the efficacy and safety of these two BIs in people with T1D (73). However, the use of CGM data in BI research has been increasing in recent years, which is in line with the overall increase in CGM usage in clinical trials. From 2013-2019, 10.7% of BI clinical trials used CGM data, which is an increase from 4.8% from 200-2006 and 8.8% from 2007-2012 (74). No major adverse events were reported, and the safety profile was in line with prior models (73).

Dexcom G6 has shown many advantages including improvements in user’s %TIR, its variety of additional features, the ability to capture more hypoglycemic events, and its FDA clearance (68, 69, 71, 73). The disadvantages of this device are similar to the MiniMed™ 780G and Guardian 4™ Sensor as they are both minimally-invasive devices that sample the interstitial fluid. This includes user discomfort due to the use of a needle and the lag-time between changes in blood glucose and device recognition of these changes in the interstitial fluid (33). Another drawback of the device is its required 2-hour warm-up period. This can be inconvenient for those who require immediate glucose readings, as they will be unable to obtain such information instantly during this time (75, 76).

### Dexcom G7

Dexcom G7 is a CGM that was cleared by the FDA on December 7, 2022 via the PMN pathway (77). In a 10.5-day non-randomized, multicenter, single-arm study of 316 adults with T1D or T2D, the overall MARD was 8.2% for arm-placed sensors and 9.1% for abdomen-placed sensors (78). The proportion of CGM values within 15% of the control values >100 mg/dL or within 15 mg/dL of control values ≤ 100 mg/dL (%15/15) as well as the %20/20 and %30/30 were reported. The control values were measured using the YSI 2300 STAT PLUS Glucose Analyzer (78). For arm-placed sensors, overall %15/15 rates were 89.6%, overall %20/20 rates were 93.2%, and %30/30 rates were 98.8%. For abdomen-placed sensors, overall %15/15 rates were 85.5%, overall %20/20 rates were 93.2%, and overall %30/30 rates were 98.1%. No major adverse events were reported during the study (78).

The Dexcom G7 offers enhancements over its predecessor, the Dexcom G6. The warmup time was shortened from 2 hours to 27 minutes, the wear length was extended from 10 days to 10.5 days, and the thickness and size of the transmitter was significantly reduced (75, 76). Dexcom G7 carries over many similar features from the Dexcom G6 such as a smartphone app, measuring glucose every 5 minutes, and sending the user smartphone alerts for aberrant glucose levels 75. Although the warm-up time has seen improvement, it is still quite long when compared to other devices that warm up in just seconds. This presents a disadvantage for users who need quick glucose measurements (79).

### FreeStyle Libre 3

FreeStyle Libre 3 is a CGM that was cleared by the FDA on May 26, 2022 via the PMN pathway (80). In a 14-day non-randomized, multicenter, single-arm study of 108 participants ≥ 6 years old with T1D or T2D, the overall MARD was 7.8% and a %20/20 rate of 93.4% compared to the control values. The control in this study was plasma venous blood glucose levels measured using the YSI 2300 STAT PLUS Glucose and Lactate Analyzer. No major adverse events were reported (81).

The advantages of FreeStyle Libre 3 are evident in its advancements over the FreeStyle Libre 2. It takes measurements every minute and transmits this data to a smartphone app. This is in contrast to the FreeStyle Libre 2, which required the user to scan the device with a smartphone to obtain glucose measurements (79, 82). This allows for the device to continuously upload data to the app and alert the user when their blood glucose is too high or too low in real-time, as opposed to the prior version which required the user to scan the device to obtain measurements (79). FreeStyle Libre 3 also has one of the lowest MARDs of available CGM devices (79). This device also has advantages in line with other CGMs, such as being minimally invasive and reducing the need for needle-sticks (79). The disadvantages of the device encompass its 1-hour warm-up time, which may be inconvenient for individuals requiring a glucose reading shortly after inserting the device (79).

### Raman spectroscopy

**Figure 2:**
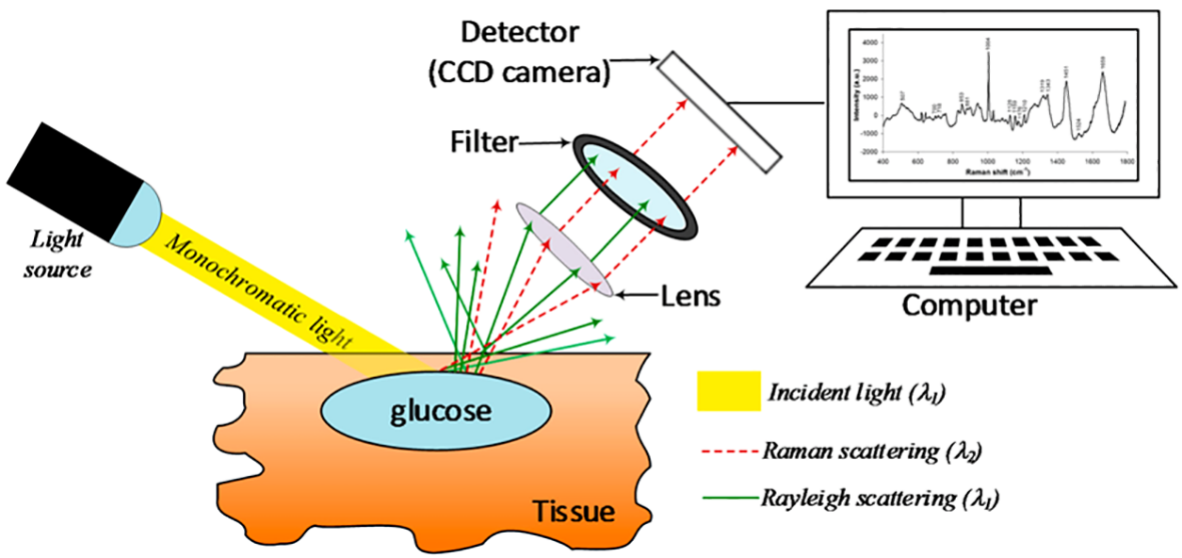
Schematic representation of a basic Raman spectroscopy instrument. Taken from Villena Gonzales (33) under the terms and conditions of the Creative Commons Attribution.

Raman spectroscopy is a non-invasive method of blood glucose measurement that uses light on the skin to vibrate glucose molecules with the resultant vibrations being used to measure blood glucose concentrations (83). The fundamental setup of a Raman spectrometer includes a lens that captures a portion of the scattered radiation and guides it to a filter, allowing only the Raman scattered light to be detected by the sensor (**Figure 2**). Research in preclinical animal models and human subjects has demonstrated that blood glucose concentrations are able to be measured from the skin using Raman spectroscopy (84, 85). Despite these findings, there have been few clinically significant devices using this technology. The C8 MediSensor was a CE-approved (Conformité Européene) device that used Raman technology, but it no longer exists due to lack of funding and was never FDA approved (83, 86). This lack of CE or FDA approved devices is due in part to challenges with accuracy and calibration stability with non-invasive devices in general (83).

Difficulties with accuracy stem from the non-invasive nature of these devices. Since they measure blood glucose concentrations indirectly, they are more susceptible to measuring physiologic variables other than glucose or having the measurement of blood glucose be disrupted by signals from external sources (87). Device calibration is another challenge faced by these devices as it typically requires frequent and lengthy processes to retain their peak accuracy (88, 89). A device that needs a high level of calibration may not have practical implications for the daily user.

However, there have been recent advances in Raman spectroscopy devices. The Raman non-invasive glucose monitor is a portable, battery-operated device with built-in safety features, WiFi capabilities, and a graphical user interface. This device uses light to measure the levels of blood glucose which is a non-invasive method of detection. The device is confocal, ensuring that the signal measured originates from the upper layers of living skin while the signal from outer layers of dead skin is suppressed. Confocality also increases the consistency of the Raman spectrum by reducing the dependency of the device-skin interface on the collected Raman signal (89).

In a clinical study, the Raman non-invasive glucose monitor was shown to be safe and maintaining calibration stability (90). However, the device was shown to have a slightly less pronounced Raman peak for darker skin colors. As the Raman peaks do not markedly differ in the thenar spectra, where the information regarding physiological glucose concentrations is found, this issue should not be of high concern (90). The device was also shown to have calibration stability by remaining stable over 15 days after the final calibration without professional stabilization. For patients with T1D, the MARD for the device is 19.9%, which is comparable to early CGM on enzyme electrodes which had a MARD between 8.8 and 19.9% (90). The advantages of this device include its safety, calibration stability, accuracy regardless of skin tone, and non-invasive nature. The disadvantages include its lack of FDA approval, the need to recalibrate after 15 days of use, and the bulky non-wearable design of the device (90).

### ZnO micropipette tips

For all glucose monitoring biosensors, electrochemical measurement is a central component that provides a highly sensitive and selective measurement of blood glucose, allowing for a wide range of detection. Additionally, it allows for the miniaturization of components, so that analysis can be performed in small volumes or even in the absence of an electrolyte. Many component materials have been tested, such as gold, silver, and platinum but such manufacturing of microelectrodes is costly (91). A cheaper alternative such as plastic is one possible substitute for the miniaturized component, most notably for micropipettes due to their cost and commercial availability.

On the other hand, other materials with more innate electrochemical detection properties, such as semiconducting metal oxides, have proven to be useful in biosensing. Zinc oxide (ZnO) has shown to have a faster electron transfer and larger reaction surface coverage. Because of these enhanced properties, a modified working electrode has been developed by growing ZnO directly on the plastic micropipettes themselves, making it a novel technique for blood glucose monitoring (91). This technique has not yet been translated to any specific CGM device; therefore, it has not been FDA approved.

**Table 3** provides a detailed summary of the clinical trials that were pivotal in securing FDA approval for various CGM systems.

**Table 3:** Key clinical trials and FDA approval milestones for continuous glucose monitoring (CGM) systems.

Device/Technology	Description of Trial	Key Findings	FDA Approval Date	Reference
Novel Modification to the Clark Enzyme Electrode	• *In vitro* experiments showing the sensor’s ability to detect physiologic glucose ranges. • *In vivo* experiments in rats showing comparable results to commercial CGMs.	N/A	N/A	Pu et al. (47)
Senseonics Eversense	• 180-day multinational, multicenter pivotal trial with 71 participants aged 18 years or older with type 1 and type 2 diabetes. • Accuracy was assessed based on comparison with venous glucose values.	• No major adverse events reported • The device has a MARD of 11.1% • The benefit of detecting aberrant glucose levels outweighs the minor adverse effects related to wearing and inserting the device	June 21, 2018	Kropff et al. (57)
MiniMed™ 780G and Guardian 4™ Sensor	• 90-day multicenter, single-arm non-randomized study of adolescents and adults. Glycemic outcomes were assessed by measuring %TIR, %TBR, %TAR, and HbA1c.	• No major adverse events reported • Minimal system exits • Improvements in %TBR • Decreased numbers of daily blood glucose measurements	March 8, 2018	Cordero et al. (61)
Dexcom G6	• 24-week multicenter, open-labeled, randomized, controlled trial of 97 adult and pediatric patients with T1D. • Dexcom G6 was compared to a sensor-augmented insulin pump. • Outcomes were measured using TIR.	• No major adverse events reported • Increased TIR by 3 hours and 21 minutes	October 26, 2018	Burnside et al. (67)
Dexcom G7	• 10.5-day non-randomized, multicenter, single-arm study of 316 adults with T1D or T2D. • Dexcom G7 was compared to venous blood glucose sampling. • Outcomes were measured using %15/15, %20/20, and %30/30, and MARD.	• No major adverse events reported • Overall MARD of 8.2% for arm-based sensors and 9.1% for abdomen-placed sensors • %15/15 of 85.5% • %20/20 of 93.2% • %30/30 of 98.1%	December 7, 2022	Garg et al. (77)
FreeStyle Libre 3	• 14-day non-randomized, multicenter, single-arm study of 108 participants aged 6 years and older with T1D and T2D. • FreeStyle Libre 3 was compared to venous blood glucose sampling. • Outcomes were measured using %20/20 and MARD.	• No major adverse events reported • Overall MARD of 7.8% • %20/20 of 93.4%	May 26, 2022	Alva et al. (80)

## Conclusion and future directions

Devices designed to continuously monitor blood glucose play a pivotal role in alleviating the burden of disease associated with T1D and represent a significant advancement in diabetes management. By providing real-time insights into glucose levels, CGM devices have transformed the way individuals with diabetes monitor and manage their condition. They offer a higher degree of freedom and control compared to traditional blood glucose testing methods, leading to improved glycemic control and quality of life for many users. Recent advancements in CGM technology, including increased accuracy, user-friendliness, and integration with insulin pumps as well as mobile devices, have further enhanced their appeal.

With the implementation of these glucose monitoring devices, individuals with T1D become empowered to learn about their condition, lifestyle modifications, treatment options, and long-term complications (22, 26, 27). Poor glycemic control can lead to retinopathy, neuropathy, and diabetic nephropathy, all of which can be avoided through meticulous monitoring of glucose levels and symptoms (7, 9–14). The real-time advantage of CGM leads to better health outcomes, both for the individuals with T1D and their providers. However, challenges remain, including the need for broader accessibility, affordability, and education to ensure that more people can benefit from this technology.

CGM devices hold a significant potential not only for managing T1D but also for T2D and gestational diabetes mellitus (GDM) (92–102). This adaptability is crucial, not only for the individual patient but also for the broader diabetes community. The expansion of CGM use into the T2D population and GDM could have several beneficial outcomes, enhancing diabetes care on multiple fronts. Firstly, the wider application of CGM devices across both T1D and T2D populations as well as for GDM can accelerate technological advancements. As demand increases, there is greater motivation for manufacturers to invest in research and development. This could lead to innovations in accuracy, user-friendliness, and integration with other health management tools. Secondly, an increase in the scale of production and adoption of CGM devices could potentially lead to a decrease in cost. Reduced prices would be particularly beneficial for individuals and healthcare systems that currently find cost a barrier to accessing advanced diabetes management tools. Furthermore, the widespread use of CGM devices in T2D and GDM care could significantly improve the availability of modern blood glucose monitoring solutions. Increased demand would likely encourage manufacturers to enhance production capabilities and distribution networks, making these devices more readily available to patients globally. CGM devices may become a standard component of diabetes care for all individuals with the condition, particularly in regions with well-developed healthcare systems. The integration of CGM devices into routine diabetes management can revolutionize care, offering real-time glucose monitoring, reducing the need for invasive finger-prick tests, and providing valuable data for more personalized treatment plans. In summary, the expansion of CGM use from T1D to T2D patients as well as for GDM represents an opportunity to advance diabetes care on a global scale. It could catalyze technological innovation, make diabetes management more cost-effective, and enhance the availability of cutting-edge monitoring tools, ultimately improving the quality of life for all patients with diabetes mellitus.

Blood glucose monitoring technologies have been expanding in recent years, especially in the area of minimally invasive and non-invasive devices (33). While there are FDA approved and cleared minimally-invasive devices, such as the Dexcom G6 and MiniMed™ 780G with Guardian™ 4 Sensor, there is still a paucity of non-invasive devices (66, 69, 73). This deficiency is not attributed to a lack of viable non-invasive technologies but rather stems from various challenges encountered in translating such technologies into functional devices for consumers and ensuring their market availability (86–88). The ongoing research and development hold the potential for the evolution of more sophisticated CGM systems in the future. These advancements may include the integration of predictive analytics and artificial intelligence, offering more personalized strategies for diabetes management. The accessibility of these advanced CGM devices is set to more effectively meet the needs of individuals having T1D, with the ultimate goal of enhancing their overall quality of life.

## Author contributions

RM: Writing – review & editing, Writing – original draft, Visualization, Validation, Supervision, Software, Resources, Project administration, Methodology, Investigation, Formal analysis, Data curation, Conceptualization. NK: Writing – review & editing, Writing – original draft, Visualization, Validation, Resources, Methodology, Investigation, Formal analysis, Data curation, Conceptualization. JM: Writing – review & editing, Writing – original draft, Visualization, Validation, Resources, Methodology, Investigation, Formal analysis, Data curation, Conceptualization. JL: Writing – review & editing, Writing – original draft, Visualization, Validation, Supervision, Software, Project administration, Methodology, Investigation, Formal analysis, Data curation, Conceptualization. KH: Writing – review & editing, Writing – original draft, Visualization, Validation, Supervision, Software, Resources, Project administration, Methodology, Investigation, Formal analysis, Data curation, Conceptualization.
