# Supplementary material for: Blood glucose monitoring devices for type 1 diabetes: a journey from the food and drug administration approval to market availability
Source: Front Endocrinol (Lausanne). 2024 Mar 15;15:1352302. doi: 10.3389/fendo.2024.1352302 (PMC10978642; doi:10.3389/fendo.2024.1352302)
Supplement: Supplementary file 1 [file DataSheet_1.pdf]

Supplementary Table 1: A Comparative Overview of the United States (U.S.) Food and Drug Administration (FDA) and European Union (EU) Regulatory Procedures

Aspect	U.S. FDA	European Union (EU)
Regulatory Framework	Regulates CGMs as medical devices, classified into Class I, II, or III based on risk.	Assessment under the EU's Medical Devices Regulation (MDR). Devices classified into classes I, IIa, IIb, and III.
Approval Process	Class III devices require Premarket Approval (PMA), Class II may require 510(k) clearance.	Requires Conformité Européenne (CE) Marking. Involves compliance with MDR standards. Notified Bodies assess devices.
Clinical Trials	Often required, extent varies based on device class and risk.	Clinical evaluations and possibly clinical investigations required, depending on device class.
Post-Market Surveillance	Mandatory to monitor device performance and safety after market entry.	Emphasis on post-market surveillance to ensure ongoing safety and performance.
Recent Developments	Updating regulatory approaches for digital health technologies and medical devices, including CGMs.	Introduction of MDR in 2017 (fully applied in 2021) brought more stringent requirements, especially in clinical evidence and monitoring.
Key Differences	Single regulatory entity (FDA). Specific pathways such as PMA and 510(k) for approval.	Multiple Notified Bodies across EU member states. More focus on compliance with MDR standards and obtaining CE marking.
